# Multi-Band Surgery for Repaired Tetralogy of Fallot Patients With Reduced Right Ventricle Ejection Fraction: A Pilot Study

**DOI:** 10.3389/fphys.2020.00198

**Published:** 2020-03-19

**Authors:** Han Yu, Pedro J. del Nido, Tal Geva, Chun Yang, Zheyang Wu, Rahul H. Rathod, Xueying Huang, Kristen L. Billiar, Dalin Tang

**Affiliations:** ^1^School of Biological Science and Medical Engineering, Southeast University, Nanjing, China; ^2^Department of Cardiac Surgery, Boston Children’s Hospital, Boston, MA, United States; ^3^Department of Surgery, Harvard Medical School, Boston, MA, United States; ^4^Department of Cardiology, Boston Children’s Hospital, Boston, MA, United States; ^5^Department of Pediatrics, Harvard Medical School, Boston, MA, United States; ^6^Mathematical Sciences Department, Worcester Polytechnic Institute, Worcester, MA, United States; ^7^School of Mathematical Sciences, Xiamen University, Xiamen, China; ^8^Department of Biomedical Engineering, Worcester Polytechnic Institute, Worcester, MA, United States

**Keywords:** tetralogy of fallot, pulmonary valve replacement, heart failure, virtual surgery, mechanical model, active contraction band

## Abstract

**Introduction:**

Right ventricle (RV) failure is one of the most common symptoms among patients with repaired tetralogy of Fallot (TOF). The current surgery treatment approach including pulmonary valve replacement (PVR) showed mixed post-surgery outcomes. A novel PVR surgical strategy using active contracting bands is proposed to improve the post-PVR outcome. In lieu of testing the risky surgical procedures on real patients, computational simulations (virtual surgery) using biomechanical ventricle models based on patient-specific cardiac magnetic resonance (CMR) data were performed to test the feasibility of the PVR procedures with active contracting bands. Different band combination and insertion options were tested to identify optimal surgery designs.

**Method:**

Cardiac magnetic resonance data were obtained from one TOF patient (male, age 23) whose informed consent was obtained. A total of 21 finite element models were constructed and solved following our established procedures to investigate the outcomes of the band insertion surgery. The non-linear anisotropic Mooney–Rivlin model was used as the material model. Five different band insertion plans were simulated (three single band models with different band locations, one model with two bands, and one model with three bands). Three band contraction ratios (10, 15, and 20%) and passive bands (0% contraction ratio) were tested. RV ejection fraction was used as the measure for cardiac function.

**Results:**

The RV ejection fraction from the three-band model with 20% contraction increased to 41.58% from the baseline of 37.38%, a 4.20% absolute improvement. The RV ejection fractions from the other four band models with 20% contraction rate were 39.70, 39.45, and 40.70% (two-band) and 39.17%, respectively. The mean RV stress and strain values from all of the 21 models showed only modest differences (5–11%).

**Conclusion:**

This pilot study demonstrated that the three-band model with 20% band contraction ratio led to 4.20% absolute improvement in the RV ejection fraction, which is considered as clinically significant. The passive elastic bands led to the reduction of the RV ejection fractions. The modeling results and surgical strategy need to be further developed and validated by a multi-patient study and animal experiments before clinical trial could become possible. Tissue regeneration techniques are needed to produce materials for the contracting bands.

## Introduction

Heart failure is a severe cardiac problem which could lead to high morbidity and mortality despite the optimal medical and electrical therapies available today (Fang et al., 2015). TOF is a common congenital heart disease. The major symptoms of TOF include ventricle septal defect, RV outflow obstruction, overriding aorta, and right ventricular hypertrophy. Currently, patients born with TOF could expect survival into adulthood after surgical repair (Bacha et al., 2001). Many long-time repaired TOF survivors are left with residual hemodynamic lesions including pulmonary regurgitation and high ventricle blood pressure, which are major causes of late-onset heart failure (Kim and Emily, 2016). The surgery for pulmonary regurgitation, PVR, showed mixed post-surgery outcomes, with some patients who recovered their RV function and had an increase in the RV ejection fraction but with some who did not (Vliegen et al., 2002; Tang et al., 2015). Del Nido proposed a scar removal and RV remodeling technique to improve post-PVR surgical outcome. In their clinical trial (NIH 5P50HL074734, Geva and del Nido), 64 patients with repaired TOF and who fulfilled the defined criteria for PVI/PVR were randomly assigned to undergo either PVI/PVR alone (*n* = 34) or PVI/PVR with surgical RV remodeling (*n* = 30). After the RV remodeling and the RV volume reduction procedures by removing, replacing, or reducing the scar and patch (non-contracting tissue) in the outflow area of the RV, the RV ejection fraction improvement was insignificant (Geva et al., 2010). Due to the complexity of the RV structure and the surgical procedures, effective PVR procedures are needed to improve the post-PVR outcome.

Computational modeling plays a vital role in cardiovascular research for better understanding of the cardiac biological mechanism and for potential clinical usage. Many investigators have made great contributions to ventricle modeling, including the Continuity package and the Physiome Project. The early cardiac stimulation model was introduced by Peskin et al. with an immersed boundary method (Peskin, 1977, 1989). A well-developed active ventricle model was introduced by McCulloch et al. (1992) and Costa et al. (1999). A novel, robust method to couple finite element (FE) models of cardiac mechanics to system models of the circulation was developed by Kerckhoffs et al. (2007). The early magnetic resonance imaging (MRI)-based mechanical ventricle models were included by Axel (2002) and Saber et al. (2001). The advancement of computational modeling made it for patient-specific ventricle models to be used in realistic applications, surgical designs, and clinical decision-making processes. Since computational models require knowledge for ventricle material properties, Sacks and Chuong (1993) and Billiar and Sacks (2000) developed biaxial mechanical testing techniques to acquire ventricle anisotropic material properties. Deng et al. (2018) used a CT-based mechanical fluid–solid interaction (FSI) ventricle model. Their results indicated that high blood pressure difference and shear stress on mitral valve leaflets might directly initiate motion in hypertrophic obstructive cardiomyopathy. Sun’s group has published extensively on quantifying myocardium and valve material properties, valve mechanics, and their impact on ventricle functions (Murdock et al., 2018; Kong et al., 2018; Sulejmani et al., 2019). We introduced a RV model for patients with TOF with a fluid–structure interaction (Tang et al., 2008; Tang et al., 2013; Yang et al., 2013; Tang et al., 2015; Yu et al., 2019). Our investigations included searching and identifying surgical options, possible factors which could improve post-PVR cardiac outcome prediction. The impact of patch size, stiffness, scar tissue removal, and RV remodeling was studied (Tang et al., 2008; Tang et al., 2013; Yang et al., 2013). Using data from computational models for 16 patients with TOF, it was found that, among the mechanical and geometrical factors considered, mechanical stress may be the best predictor for post-PVR cardiac outcome (Tang et al., 2015). Yu et al. (2019) used patient-specific CMR data from six healthy volunteers and 16 TOF patients and estimated the RV material stiffness parameter values. Their results indicated that the mean end-ejection effective YM value of TOF patients was 78.6% higher than that of the healthy group (1,952 vs. 1,093 kPa, *p* = 0.016). The mean end-ejection YM value from the worse-outcome TOF group was 59.5% higher than that from the better-outcome TOF group (2,400 vs. 1504 kPa, *p* = 0.008). Active contracting band insertion models using one band were also introduced using CMR data from one TOF patient to investigate the effect of band material stiffness variations, band length, and active contraction (Yang et al., 2013). The initial results were promising.

In this paper, we continue our effort to test the feasibility of the novel PVR surgical procedures using active contracting bands to improve the post-PVR cardiac outcome. A total of 21 models combining five different band insertion options with passive and active bands and three different contraction ratios were constructed using CMR data from one repaired TOF patient to find the potential possible benefit of active contracting bands.

## Materials and Methods

### Data Acquisition

**FIGURE 1:**
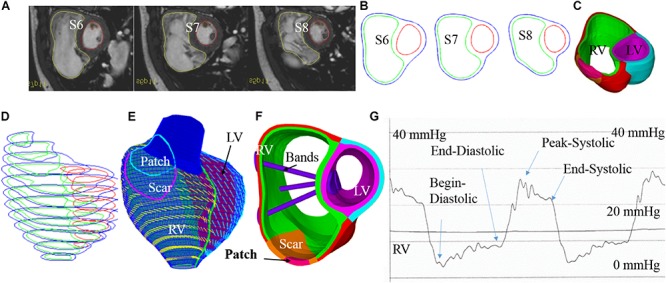
Cardiac magnetic resonance-based model construction process and pressure conditions. **(A)** Selected CMR slices from a patient, end of systole. **(B)** Segmented contours. **(C)** Two-layer structure. **(D)** Zero-load geometry. **(E)** Model with fiber orientations. **(F)** Ventricle with three bands inserted. **(G)** Recorded RV pressure profile. This figure was made with components from Figure 1 in Yu et al. (2019) with copyright held under a creative commons license.

Cardiac magnetic resonance data were provided by the Boston Children’s Hospital from their clinical RV surgical remodeling trial (NIH 5P50HL074734, Geva and delNido). Written informed consent was obtained from the participants. The CMR image was acquired from one TOF patient (age, 22.5 years; gender, male) before and after PVR surgery using electrocardiography-gated, breath-hold, steady-state, cine precession MRI (slice thickness: 6–8 mm, interslice gap: 0–2 mm, 30 frames per cardiac cycle). The patch, scar, and valve were located based on cine MRI, flow data, and delayed enhancement CMR and further verified by the surgeon (PJdN, over 30 years of experience) who performed the PVR surgery. The endocardial and epicardial borders were traced manually and analyzed using commercially available software QMass (QMass, Medis Medical Imaging Systems, Leiden, Netherlands) for the entire cardiac cycle containing 30 time points, each with a 3D CMR data set. Simpson’s method was applied to calculate the end-diastolic volume (EDV), the end-systolic volume (ESV), the stroke volume, and the ejection fraction. Blood pressure in the RV was measured via cardiac catheterization procedures. Figure 1 shows the selected CMR slices together with segmented contour plots, zero-load ventricle geometries, reconstructed 3D RV/LV geometry with scar, patch and myocardium fiber orientation, and measured ventricle pressure conditions from the patient. The details about our pre-shrinking process to obtain zero-load geometry and 3D geometry reconstruction procedures are given in the “Governing Equations for Our RV/LV/Patch/Band Models” section. The demographic data are given in Table 1.

**TABLE 1:** Demographic data of the TOF patient.

Age (year)	22.5
Gender	Male
Heart rate (beats/min)	56
Maximum RV pressure (mmHg)	31.4
Minimum RV pressure (mmHg)	2.16
RV EDV (ml)	406.9
RV ESV (ml)	254.5
Pre-op RV EF (%)	37.46
RV SV (ml)	152.4

### Novel PVR Surgical Strategy Using Active Contracting Band and Feasibility Study Using Band Models

**FIGURE 2:**
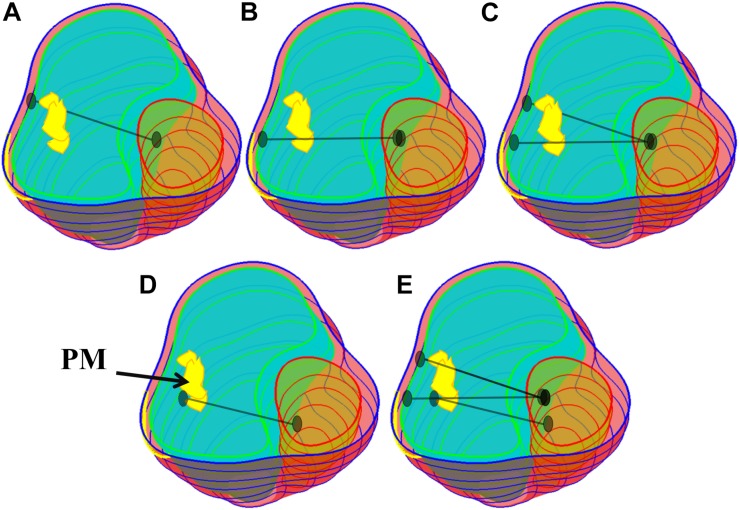
Five band insertion surgical plans. **(A)** Plan A: A band at anterior to the middle of PM. **(B)** Plan B: A band at posterior to the middle of PM. **(C)** Plan C: Double bands, plans A and B combined. **(D)** Plan D: A band at the base of the PM. **(E)** Plan E: Combination of plans C and D. PM, papillary muscle. Colors used: light blue, RV inner surface; green, LV inner surface; yellow, papillary muscle; black, band.

One major reason why TOF patients have poor RV cardiac function performance (normally measured by ejection fraction) is the weakened contractility of their RV tissue. We hypothesized that adding active contracting band(s) to the ventricle could improve its contractility and cardiac function. We are using our modeling approach to test the feasibility of this hypothesis. A total of 21 computational band models based on patient-specific CMR data with different band designs (location, number of bands, and percent of contraction rate) were constructed to investigate the impact of band insertion surgery. These models included five different band insertion plans (plans A–E, Figure 2) combined with passive elastic bands and active contracting bands with three different band contraction ratios (10, 15, and 20% band zero-stress length reduction). Plan A has a band at the anterior to the middle of the papillary muscle (PM). Plan B uses a band at the posterior to the middle of the PM. Plan C has two bands with plans A and B combined. Plan D has a band at the base of the PM. Plan E is a three-band model with all the three single bands combined. A summary of the band models is given in Table 2.

**TABLE 2:** Band model summary, band location and numbers, contraction ratios, and zero-load band length for all 21 RV/LV models.

**Models**	**Plan**	**Band no.**	**Contraction ratio**	**Zero-load band length (cm)**	**Band location**
Baseline	–	0	–	–	–

A000	A	1	Passive	3.94 (100%L)	Anterior to the middle of PM
A010	A	1	10%	3.54 (90%L)	Anterior to the middle of PM
A015	A	1	15%	3.35 (85%L)	Anterior to the middle of PM
A020	A	1	20%	3.15 (80%L)	Anterior to the middle of PM

B000	B	1	Passive	4.02 (100%L)	Posterior to the middle of PM
B010	B	1	10%	3.61 (90%L)	Posterior to the middle of PM
B015	B	1	15%	3.41 (85%L)	Posterior to the middle of PM
B020	B	1	20%	3.21 (80%L)	Posterior to the middle of PM

C000	C	2	Passive	3.94, 4.02 (100%L)	Plan A and B combined
C010	C	2	10%	3.54, 3.61 (90%L)	Plan A and B combined
C015	C	2	15%	3.35, 3.41 (85%L)	Plan A and B combined
C020	C	2	20%	3.15, 3.21 (80%L)	Plan A and B combined

D000	D	1	Passive	3.16 (100%L)	The base of the PM
D010	D	1	10%	2.85 (90%L)	The base of the PM
D015	D	1	15%	2.69 (85%L)	The base of the PM
D020	D	1	20%	2.52 (80%L)	The base of the PM

E000	E	3	Passive	3.94, 4.02, and 3.16 (100%L)	Combination of plan C and D
E010	E	3	10%	3.54, 3.61, and 2.85 (90%L)	Combination of plan C and D
E015	E	3	15%	3.35, 3.41, and 2.69 (85%L)	Combination of plan C and D
E020	E	3	20%	3.15, 3.21, and 2.52 (80%L)	Combination of plan C and D

*PM, papillary muscle.*

**FIGURE 3:**
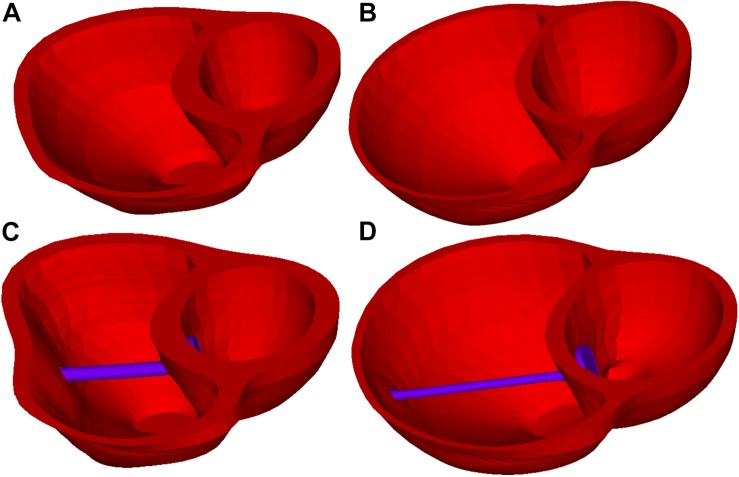
Right ventricle /LV/band models at end-ejection (minimum RV volume) and end-filling (maximum RV volume). **(A)** Baseline model at end-ejection. **(B)** Baseline model at end-filling. **(C)** Model D020 (active band model) at end-ejection. **(D)** Model D020 (active band model) at end-filling.

The idea of passive and active bands models was introduced by Dr. del Nido and a paper with several one-band models was published (Yang et al., 2013). The zero-stress length (denoted by *L*) of the passive band was equal to the distance between the two locations on the ventricle wall from the zero-load no-band model where the band would be placed (100% of *L*). All active contracting bands had *L* as their “relaxed” zero-stress length and shortened to their contracted zero-stress length by 90, 85, and 80% of *L*, respectively. Due to their ability to actively shorten and relax, they can help the ventricle to contract during systole phase but would not resist the ventricle’s relaxation in the diastolic phase (see Figure 3). The active contracting bands would contract and relax with the ventricle, which means that the zero-stress band length and the material parameters would change with ventricle contraction and relaxation.

This paper mainly aims to demonstrate the potential benefit of actively contracting bands if such materials could be made available. In our modeling process of the actively contracting band, the zero-stress band length was 80, 85, or 90% *L* in the contracted state of the band, corresponding to the contraction ratio of 20, 15, or 10%, respectively. At the end of the diastole phase, the zero-stress band length would be relaxed to *L*. In addition to active contraction and relaxation, the band would also have elastic expansion and contractions, in conjunction with the ventricle contractions and expansions.

### Governing Equations for Our RV/LV/Patch/Band Models

For simplicity and efficiency in the model construction cost, structure-only models were used in this paper, which are sufficient to simulate and quantify the ventricle deformation and volume changes. Our models included RV, LV, patch (with RV outflow track and scar), and bands, so the name RV/LV/patch/band may be used for convenience when proper. Both the RV and the LV were included since it is difficult to keep the RV in its shape without the support of the LV or the artificially imposed constraints. Scar tissue and patch were included and whose shape and location were carefully reviewed by the surgeon (PN) and the radiologist (TG). The models with different band numbers and locations were considered. Ventricle tissue (myocardium) was assumed to be hyperelastic, anisotropic, nearly incompressible, and homogeneous. The patch, scar, and band materials were assumed to be hyperelastic, isotropic, nearly incompressible, and homogeneous. The non-linear Mooney–Rivlin model was used to describe the non-linear anisotropic and isotropic material properties (Yang et al., 2013; Yu et al., 2018). The measured right and LV blood pressure values were implemented on the right and the LV inner surfaces, respectively. The governing equations for our RV/LV/patch/band models were given by Tang et al. (2008):

(1) $$\rho \,v_{i,t\,t}=\sigma_{i\,j,j},i,j=1,2,3;\text{sum over}\,j,$$

(2) $$\varepsilon_{i\,j}=\frac{1}{2}\left(v_{i,j}+v_{j,i}+v_{\alpha ,i}v_{\alpha ,j}\right),i,j,=1,2,3,$$

(3) $${p|}_{R\,V}=p_{R\,V}\,\left(t\right),{p|}_{L\,V}=p_{L\,V}\,\left(t\right)$$

**FIGURE 4:**
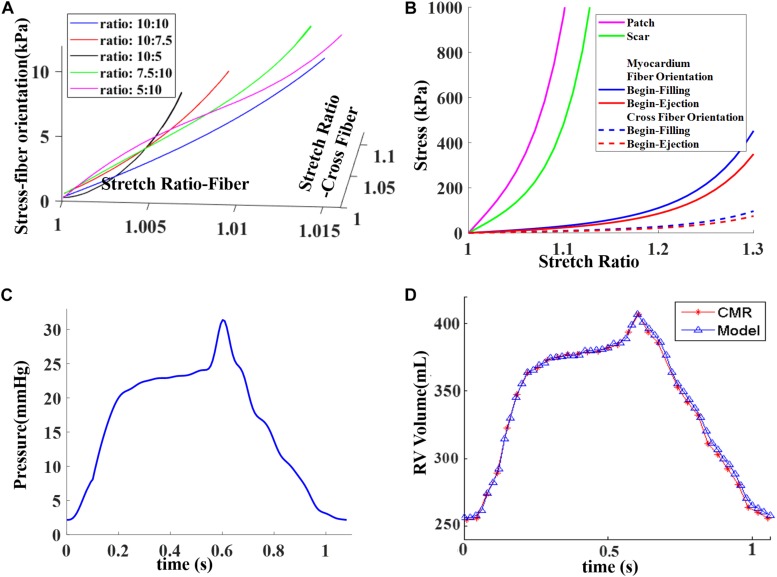
Material stress–stretch curves and pressure conditions used in the paper and computational RV volume curve matching CMR-measured data. **(A)** Stress–stretch curves from Mooney–Rivlin models fitting data from biaxial test in fiber orientation. Five curves for five different stress ratios (stress in fiber direction vs. stress in cross-fiber direction) were plotted. **(B)** Stress–stretch curves for patch, scar, and myocardium in both fiber and cross-fiber directions for comparison purpose. Myocardium fiber and cross-fiber curves were made using equal stretch ratios in both fiber and cross-fiber directions. **(C)** Blood pressure in RV chamber. **(D)** Computed RV volume from baseline model matching CMR-measured volume. **(C)** was taken from Figure E1 C in Yang et al. (2013), with permission.

The isotropic Mooney–Rivlin strain energy function is given by:

(4) $$W=c_{1}\,\left(I_{1}-3\right)+c_{2}\,\left(I_{2}-3\right)+D_{1}\,\left[\exp\left(D_{2}\,\left(I_{1}-3\right)\right)-1\right]$$

where *I*_1_ and *I*_2_ are the first and second strain invariants given by:

(5) $$I_{1}=\sum C_{i\,i},I_{2}=\frac{1}{2}\,\left(I_{1}^{2}-C_{i\,j}\,C_{i\,j}\right)$$

where *C* = [*C*_*ij*_] = *X^*T*^X* is the right Cauchy–Green deformation tensor, *X* = [*Xij*] = ∂[*x*/*a*, (*x*_*i*_) is the current position,(*a*_*i*_) is the original position, and *c*_*i*_ and *D*_*i*_ are the material parameters chosen to match the experimental or the patient-specific CMR measurements. The strain energy function for the anisotropic modified Mooney–Rivlin model was obtained by adding an additional anisotropic term in Eq. (4) (Tang et al., 2008):

(6) $$W=c_{1}\,\left(I_{1}-3\right)+c_{2}\,\left(I_{2}-3\right)+D_{1}\,\left[\exp\left(D_{2}\,\left(I_{1}-3\right)\right)-1\right]+\frac{K_{1}}{K_{2}}\,\left[\exp\,{\left(I_{4}-1\right)}^{2}-1\right]$$

where *I*_4_ = *C_*ij*_(n_*f*_)_*i*_(n_*f*_)_*j*_, C_*ij*_* is the Cauchy–Green deformation tensor, *n*_*f*_ is the fiber direction, and *K*_1_ and *K*_2_ are the material constants. With the parameters well chosen, the modified Mooney–Rivlin model could fit the direct measurement of the experiment stress–strain data from our biaxial test on the myocardium (Yu et al., 2018). The material parameter values are given in Table 3. The ventricle material parameter values were determined to fit the CMR-measured volume data. Stress–stretch curves are shown in Figure 4. It should be noted that both the patch and the scar materials were assumed to be isotropic. In Figure 4B, the myocardium fiber and the cross-fiber curves were made using equal stretch ratios in both the fiber and the cross-fiber directions so that it was possible to make 2D stress–stretch curves.

**TABLE 3:** Summary of the parameter values of the Mooney–Rivlin models for all materials, isotropic and anisotropic models (*c*_2_ = 0 kPa).

**Material/model**	**c_1_ (kPa)**	**D_1_ (kPa)**	**D_2_**	**K_1_ (kPa)**	**K_2_**
Patch (isotropic)	38.45	38.45	9.0	0	–
Scar (isotropic)	19.23	19.23	9.0	0	–
**Myocardium, end-ejection**					
RV/LV inner layer	3.82	1.20	3.0	18.28	3.0
RV/LV outer layer	4.36	1.12	3.0	17.76	3.2
**Myocardium, end-filling**					
RV/LV inner layer	2.95	0.93	3.0	14.12	3.0
RV/LV outer layer	3.37	0.87	3.0	13.72	3.2

**FIGURE 5:**
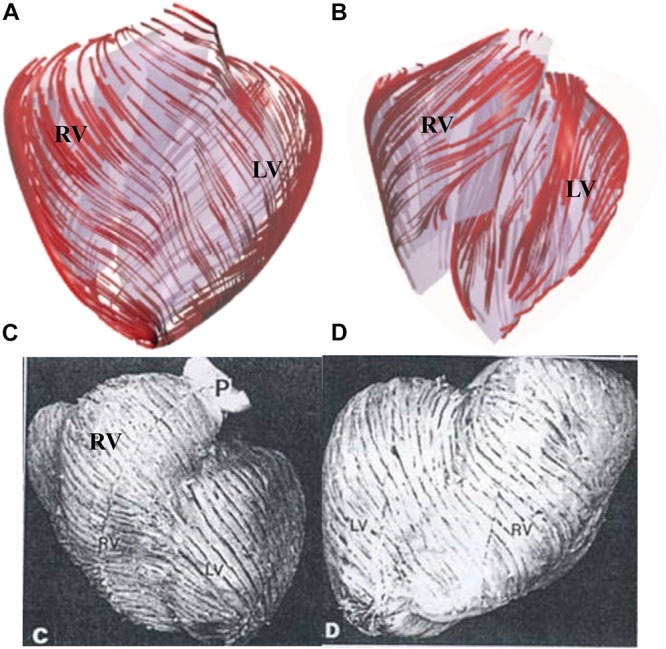
Ventricle fiber orientation. **(A)** Pig ventricle: epicardium. **(B)** Pig ventricle: endocardium. **(C)** Human heart from a TOF patient: front view. **(D)** Human heart, back view. This figure was made with components from Figures 3A–D in Yang et al. (2013), with permission.

The orientation of the myofibrils caused the myocardial tissue to exhibit mechanical anisotropy. Since patient-specific fiber orientation data were not available, we chose to construct a two-layer RV/LV model and set fiber orientation angles using the fiber angles published by Sanchez-Quintana et al. (1996), Nash and Hunter (2000), and Hunter et al. (2003) and available data in human. In our two-layer models, the left ventricular fiber orientation was approximately −60° (relative to the circumferential direction) at the outer layer and +80° at the inner layer. The RV fiber orientation was set at −45° at the outer layer and at +40° at the inner layer (see Figures 1, 5).

### Pre-shrink Process and Myocardium Material Parameters for Patient-Specific CMR-Based Model

Under *in vivo* conditions, the left and the RVs are under pressure and their zero-load ventricular geometries were unknown from *in vivo* CMR images. Thus, in our model construction process, a pre-shrink process was applied to the *in vivo* begin-diastole ventricular geometries to generate the zero-stress geometry for the computational simulation so that, after the pressure was applied, the ventricle could approximately regain its *in vivo* morphology. Shrinking is achieved by shrinking each slice (short-axis direction) with a short-axis shrinking rate and by reducing the slice distances (long-axis direction) with a long-axis shrinking rate. The shrinkage for the inner slice contour was 2–3% based on the RV EDV, RV ESV, and blood pressure. The outer slice contour shrinkage was determined to conserve the total ventricular wall mass. The outer slice contour shrinkage was smaller than the inner slice contour to keep the ventricle tissue total volume (mass) conserved. After the pre-shrink process was implemented, the parameters in Eq. (6) for myocardium were adjusted iteratively until a good agreement between the computational and the CMR-measured volume data was found (error <0.2%). The details were described in Tang et al. (2008, 2013, 2015), Yang et al. (2013), and Yu et al. (2018).

### Solution Methods, Data Extraction, and Model List

The 21-band models were constructed and solved by ADINA (ADINA R&D, Watertown, MA, United States) using unstructured finite elements and the Newton–Raphson iteration method. The simulation procedures were continued until the differences in the solutions between the last two periods became less than 0.1%. Our experience indicated that three periods were enough (solutions for periods 2 and 3 were almost identical). The solutions for the last period were accepted for analysis. The maximum principal stress and strain (both are scaler functions) were used as the representative stress/strain variables. The maximum principal stress and strain values from all RV inner surface points (100 evenly spaced points from each slice of the RV inner surface) were extracted from the model and their mean values at begin-filling (BF) and begin-ejection (BE) were recorded for comparison. The RV stress, strain, and pressure reach their minimum and maximum in a cardiac cycle, respectively.

Mesh analyses were performed for each model so that the solutions became stable and mesh-independent. The mesh analysis was accomplished by decreasing the mesh size until the solution differences in stress/strain from two consecutive meshes were less than 1%. The RV stress and strain errors from one sample model with different element numbers are given in Table 4. Stress and strain errors were defined by L_1_-norm errors between solutions from two consecutive meshes. The details were described in Yu et al. (2019).

**TABLE 4:** Stress and strain errors from models with different mesh numbers.

**Element number**	**Stress error (%)**	**Strain error (%)**
8451	8.66	10.19
12470	4.80	3.64
16956	3.54	2.45
20630	2.50	2.39
25550	1.33	1.66
30554	0.98	0.81

The RV ejection fraction was used as the index (marker) for evaluating ventricle cardiac function. The difference between the pre-operation and the post-operation RV ejection fraction, ΔEF, was used to qualify post-PVR surgery cardiac function improvement:

(7) Δ⁢EF=Post-PVR EF-Pre-PVR EF.

Pre-PVR no-band RV/LV model was constructed and used as the baseline model. Models “AXXX,” “BXXX” to “EXXX” corresponded to the five band insertion surgery plans with four active contraction ratios of 0, 10, 15, and 20%, respectively, where “XXX” represented the band contraction ratio and “000” represented the RV/LV models with no-contraction passive bands. The simulation results are presented in the “Results” section.

## Results

The analyses of the 4D (time +3D) ventricle stress and strain data are overly complicated. Data at two critical time points, i.e. begin-filling and begin-ejection, were selected for analyses and comparative studies. The RV begin-filling and begin-ejection volume, ejection fraction, ejection fraction change from pre-PVR to post-PVR (ΔEF), and mean stress and strain values are given in Table 5. For the patient under consideration, the RV ejection fraction from our baseline no-band model was 37.38%, agreeing well with the EF data (37.46%) from direct CMR measurement.

**TABLE 5:** Right ventricle ejection fraction and wall stress/strain data from 21 models: five surgery plans combined with passive and active bands of three different contraction ratios and a RV/LV model with no band.

**Models**	**Begin-filling**			**Begin-ejection**			**EF (%)**	**ΔEF (%)**
			
	**RV volume (ml)**	**Stress (kPa)**	**Strain**	**RV volume (ml)**	**Stress (kPa)**	**Strain**		
Baseline	254.74	3.05	0.031	406.80	64.21	0.288	37.38	–
A000	253.19	2.96	0.031	397.95	60.97	0.288	36.38	−1.00
A010	249.29	3.02	0.030	406.35	61.72	0.291	38.65	1.27
A015	247.16	3.08	0.031	406.27	61.68	0.291	39.16	1.78
A020	244.90	3.17	0.033	406.15	61.64	0.291	39.70	2.32
B000	253.63	3.01	0.031	397.02	60.74	0.285	36.12	−1.26
B010	250.40	3.07	0.031	409.08	64.72	0.291	38.79	1.41
B015	248.44	3.10	0.031	407.15	64.72	0.285	38.98	1.60
B020	246.47	3.16	0.032	407.07	64.72	0.285	39.45	2.07
C000	252.30	2.83	0.029	386.57	57.00	0.274	34.73	−2.65
C010	246.80	2.93	0.029	405.09	63.17	0.287	39.07	1.69
C015	243.81	3.05	0.031	405.96	63.92	0.289	39.94	2.56
C020	240.64	3.21	0.033	405.83	63.98	0.289	40.70	3.32
D000	250.80	2.74	0.030	383.79	55.25	0.284	34.65	−2.73
D010	247.52	2.68	0.029	402.73	63.56	0.302	38.54	1.16
D015	245.60	2.73	0.029	402.79	63.67	0.301	39.03	1.65
D020	243.71	2.92	0.031	400.63	64.86	0.306	39.17	1.79
E000	248.83	2.82	0.028	375.78	54.60	0.278	33.78	−3.60
E010	242.37	3.01	0.030	402.81	67.33	0.301	39.83	2.45
E015	238.86	3.18	0.032	402.66	67.47	0.301	40.68	3.30
E020	235.13	3.39	0.035	402.45	67.76	0.302	41.58	4.20

### Passive Band Had Negative Impact on Right Ventricle Function

Initially, one might think that an elastic band may be able to help the ventricle to contract and improve the ejection fraction. That was proven not true for a reason we did not think of: while it could be true that the band would help the ventricle to contract during systole by its elastic contraction, the band would also be holding the ventricle during diastole and resisting its expansion. The ventricle would not be able to reach its no-band maximum end-diastiole volume.

Our results indicated that the ejection fractions from those passive band models had significant decreases after the passive bands were inserted. The ΔEF for the passive band models A000, B000, C000, D000, and E000 were −1.00, −1.26, −2.65, −2.73, and −3.60%, respectively. The ejection fraction losses were caused by the decrease of the RV begin-ejection volume (the same as the end-diastole volume). The begin-ejection RV volume of the passive band models was 2.2, 2.5, 5.2, 6.0, and 8.25% lower than that from the baseline model. The baseline begin-ejection RV volume was 406.80 ml, while the band model begin-ejection volumes were A000—397.95 ml, B000—397.02 ml, C000—386.57 ml, D000—383.79 ml, and E000—375.78 ml.

### Active Contractional Bands Improved the Right Ventricle Ejection Fraction

Results shown in indicated that plan E (three bands) with a band contraction ratio of 20% (model E020) improved the RV ejection fraction to 41.59%, which represented 4.20% absolute improvement or 11.24% relative improvement compared to the baseline EF value. The ejection fractions for plans A–D with band contraction ratios of 20% were 39.70, 39.45, 40.70, and 39.17%, respectively. It was clear that plan E with three bands had the best EF improvement, and plan C with two bands had the second best EF improvement. Plans A, B, and D (single with different locations) had similar EF improvements. Plan A was slightly better in general among the three (except B010) because the band location was more balanced.

**FIGURE 6:**
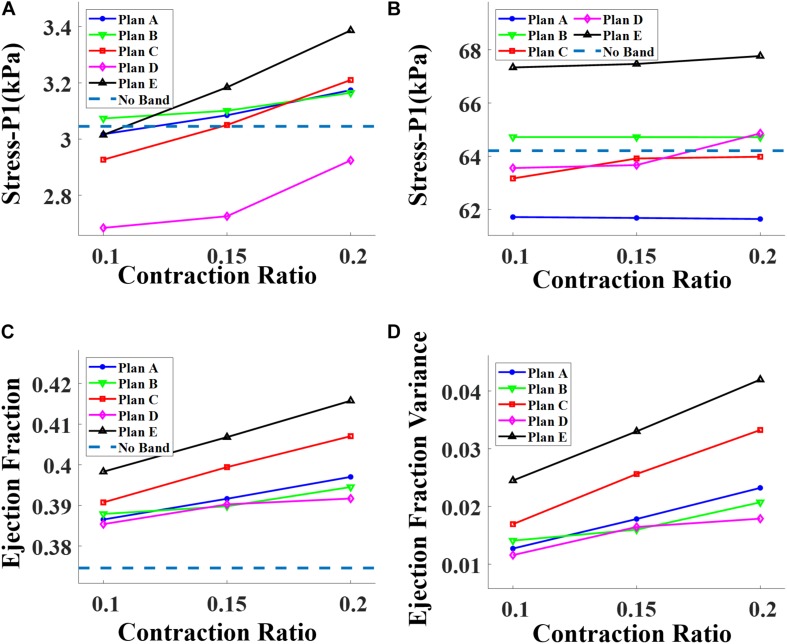
Impact of contraction ratio on RV wall stress, ejection fraction, and ejection fraction improvement with band insertion surgery. **(A)** Begin-filling stress. **(B)** Begin-ejection stress. **(C)** Ejection fraction. **(D)** Ejection fraction improvement.

Three different contraction ratios were applied on the band: 10, 15, and 20%. The surgeries with a band contraction ratio of 20% had the best improvement on EF, as expected. The same relations were also found among the models of surgery plans A, B, C, and D. The ploy-lines/bar plots of RV stress and EF vs. band contraction ratio are shown in Figure 6.

### Band Location Could Lead to Different RV Stress and Strain Distributions

The ventricle stress and strain variance caused by the inserted bands was small and insignificant. Table 5 shows that the RV mean stress values (unit in kPa) at begin-ejection from the five band models (A020–E020) with 20% band contraction ratio were 61.64, 64.72, 63.98, 64.86, and 67.76 kPa, respectively, compared to the baseline mean stress value at 64.21 kPa. The RV mean strain values at begin-ejection from the five band models (A020–E020) with 20% band contraction ratio were 0.291, 0.285, 0.289, 0.306, and 0.302 compared to the baseline mean stress value at 0.288. The RV mean stress and strain values at begin-filling were small and their relative differences were also small. Overall the band insertion did not cause differences to the RV mean stress/strain values.

**FIGURE 7:**
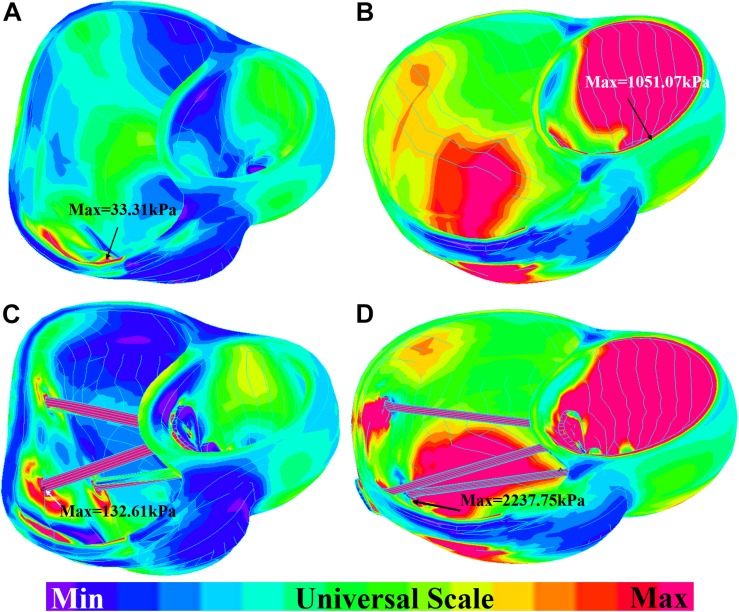
Short-axis view: ventricle stress plots (stress-P_1_) of baseline model and model E020. **(A)** Baseline model at begin-filling. **(B)** Baseline model at begin-ejection. **(C)** Model E020 at begin-filling. **(D)** Model E020 at begin-ejection.

**FIGURE 8:**
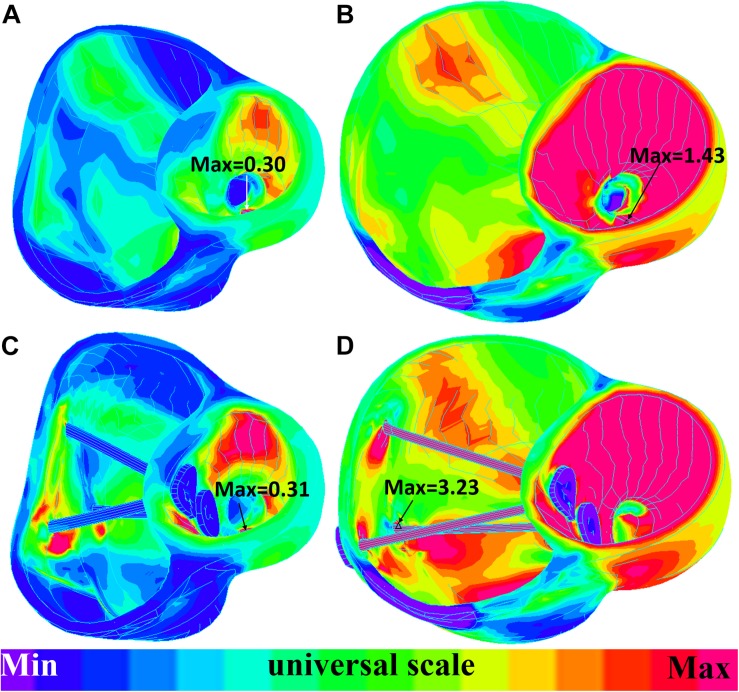
Short-axis view: ventricle strain plots (strain-P_1_) of baseline model and model E020. **(A)** Model E020 at begin-filling. **(B)** Model E020 at begin-ejection. **(C)** Baseline model at begin-filling. **(D)** Baseline model at begin-ejection.

**FIGURE 9:**
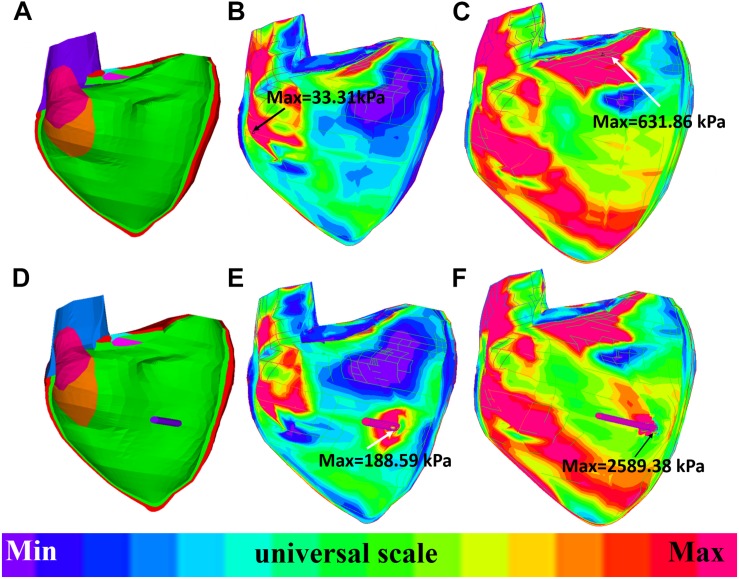
Long-axis cut-surface: stress-P_1_ band plots of baseline model and model B020. **(A)** Zero-load geometry of baseline model. **(B)** Baseline model at begin-filling. **(C)** Baseline model at begin-ejection. **(D)** Zero-load geometry of model B020. **(E)** Model B020 at begin-filling. **(F)** Model B020 at begin-ejection.

The impact of band insertion can be better observed by the stress/strain local distributions. Figures 7–9 give the stress number and locations. The local stress/strain behaviors can be important band surgical suture considerations.

## Discussion

### PVR Surgical Options and Challenge for Band Insertion Strategy

While PVR surgery has to be performed for repaired TOF patients in their lives, improving post-PVR cardiac function remains a challenge due to the complexities in surgical procedures, patch material, RV geometry, and remodeling process after PVR. We have demonstrated that band insertion with active contracting bands has good potential for possible improvements. Our modeling study showed that active contractional bands could improve the RV ejection fraction from 37.38 to 41.58% for the patient studied. This is an absolute improvement of 4.20% or a relative improvement of 11.24%. This compares favorably with the published drug trials to treat heart failure in which an improvement of 3 to 4% in LVEF resulted in significant improvement in functional capacity (Aleksova et al., 2012). Combined with scar trimming, patch optimization, and RV remodeling in PVR surgery, the band insertion technique may have further improvement in post-PVR cardiac function.

As we tried to demonstrate the feasibility of the band insertion strategies, the availability of the active contracting bands is an issue. Myocardium tissue regeneration has been an active research area in recent years. Ideally, the active contraction bands would be made of myocardium which could contract synchronized with the ventricle contraction. Currently, one viable solution is to use stem cells and biomaterial to generate an active contraction band made of contractional filament. Huge efforts had been devoted into cardiac repair and regeneration with biomaterial scaffolds such as fibrin or collagen (Barsotti et al., 2011; Cui et al., 2016). Proulx et al. (2011) described a band made of fibrin that could be seeded with mesenchymal stem cells and stitched through a collagen gel. A contracting band may be made by seeding these fibrin bands with contractile cells or stem cells. The details were discussed in our previous work (Tang et al., 2013).

Like many popular commercial mechanical ventricle assist devices for ventricular heart failure such as Impella RP (Abiomed, Danvers, MA, United States), HeartMate II (St. Jude Medical, Minneapolis, MN, United States) or HeartWare HVAD (HeartWare, Framingham, MA, United States), active contractional bands aim to help the ventricle to contract and push more blood to flow into the aorta or the pulmonary artery. These commercial ventricle assist devices have made great contributions to heart failure treatment with massive clinical cases (Sabashnikov et al., 2014; Anderson et al., 2015). RV heart failure has become a major clinical problem for TOF patients as the surgeries greatly extended their life expectancy (Wald et al., 2015). However, ventricle assist devices have not gained widespread use in adult patients with RV heart failure caused by a congenital heart disease due to many potential barriers (Everitt et al., 2015; Wald et al., 2015). Instead of using a pump draining blood from the ventricle and delivering it to the pulmonary artery or the artery directly, active contractional band insertion surgery presents an innovative method by helping the ventricle in squeezing more blood into the pulmonary artery.

### Optimization of Band Contraction Ratio, Number, and Location

Reduction of RV ejection fraction with elastic passive bands indicated that passive band surgery might not be an optimal surgery treatment for TOF patients. The PVR surgery with more bands and higher active contraction ratios had better improvement in recovering the RV function. However, in our work, the active band contraction ratios (10, 15, and 20%) were only our hypothetical numbers. An early theoretical analysis by Gordon et al. (1966) indicated that the contraction ratio of the sarcomere could reach about 40%. With higher contraction ratio, greater improvements in the RV ejection fraction is possible. In order to test the band performance under an extreme condition of 40% band zero-load contraction ratio, an experiment was conducted, showing that the ejection fraction of the RV model after surgery plan E could reach 45.33%, providing a 7.87% absolute improvement. The ejection fractions of plans A, B, C, and D were 42.78, 41.83, 44.15, and 42.10%, respectively. However, normally, the myocardium sarcomere active contraction ratio could hardly be so extreme. This experiment simply aimed to explore the maximum ability of the band to improve the RV function. It should be noted that the band contraction should be consistent with the ventricle contraction. Meanwhile, the RV stress of plan E and the 40% band active contraction was 32.13% higher than that of the baseline. The results are shown in Table 6. Extreme band contraction could cause damage to the ventricle wall and defeat our purpose. Compared to plans A and B, plan D had much lower RV stress values and their differences in △EF were small. Thus, it is reasonable to believe that plan D may be the optimal surgery plan if only one band was used in the surgery. The RV stress values of model E010 was below the base value and most of the band models and yet its RV ejection fraction improvement was higher than model C010 and all the models with one band, from which a hypothesis can be drawn: multi-band surgeries with low band contraction ratios could have better performance than single-band surgeries with higher band contraction ratios.

**TABLE 6:** Right ventricle EF and wall stress/strain from models of band active contraction ratio of 40%.

**Surgery plan**	**Begin-filling**			**Begin-Ejection**			**EF (%)**	**ΔEF (%)**
	**RV volume (ml)**	**Stress (kPa)**	**Strain**	**RV volume (ml)**	**Stress (kPa)**	**Strain**		
A	233.76	3.62	0.038	408.59	60.61	0.29	42.79	5.33
B	236.44	3.40	0.039	406.45	64.18	0.28	41.83	4.37
C	225.76	3.96	0.045	404.21	65.13	0.29	44.15	6.69
D	230.04	3.19	0.035	397.34	87.45	0.31	42.11	4.65
E	217.96	4.03	0.046	398.68	68.84	0.30	45.33	7.87
								

At least, under the current circumstances, the passive bands could increase the RV stress and decrease the RV EF, causing damage to the ventricle wall and a negative effect on the RV function. For active band surgery plans, plan E (three-band model) with an active band contraction ratio of 20% had the best performance on improving the RV EF.

### Model Validation

Model validation is a key step for all modeling work. For our modeling effort, validation takes several stages. The first stage is validation of our no-band model using available *in vivo* MRI-measured RV volume. The RV/LV models were constructed with material parameters chosen to match patient-specific RV volume data. With this patient-specific RV/LV model, the band models were constructed (by adding bands to the validated RV/LV models) to conduct our feasibility study. The validation of these band models would require results and data from patients who would have received those surgeries with contracting bands. Those data are currently not available. In fact, this feasibility study is aimed to obtain pilot data to support further effort in investigations in this direction, including tissue regeneration effort to produce the contracting band and actual surgical experimentation using animal models (pigs). The purpose of using computer-simulated virtual surgeries is to avoid direct experimentation on patients. That is the value of our approach.

### Model Limitations and Future Directions

Several limitations should be acknowledged in our modeling study: (a) only one patient’s data were used in this study. The band insertion benefits may vary from patient to patient. A multi-patient study should be conducted to help us draw more valid conclusions and further verify and confirm our findings; (b) the fluid–structure interaction should be included to obtain both blood flow and myocardium stress and strain data; (c) both the tricuspid valve and the pulmonary valve mechanics were not included; (d) data shown in Table 7 indicate that the RV stress and strain values could be affected by myocardium fiber orientations. Patient-specific TOF RV/LV myocardium fiber orientations should be acquired and used in our future models; (e) the pre-shrink method applied to *in vivo* ventricle geometry obtained from CMR data to obtain approximate zero-load geometries could alter the shape of the ventricle when *in vivo* pressure was applied; (f) band stress and strain data were not available since the variance of zero-load length during band contraction and relaxation was not considered; (g) in our stimulation, the band did not change the RV blood pressure; and (h) in our future study, the band material parameters will be identical to the healthy myocardium as described in Yu et al. (2018).

**TABLE 7:** Impact of myocardium fiber orientation RV stress and strain values.

**RV fiber**		**Inner layer: 160° Outer layer: 75°**	**Inner layer: 100° Outer layer: 15°**	**Inner layer: 40° Outer layer: −45°**
Begin-filling	Stress (kPa)	3.27	3.13	3.05
	Strain	0.032	0.033	0.031
Begin-ejection	Stress (kPa)	73.96	61.03	64.21
	Strain	0.295	0.307	0.288

## Data Availability Statement

All datasets generated for this study are included in the article/supplementary material.

## Ethics Statement

Written informed consent was obtained from the individual(s) for the publication of any potentially identifiable images or data included in this article.

## Author Contributions

PN, TG, and RR collected the data. HY, CY, ZW, XH, and DT computed the modeling and analyzed the results. KB provided the biaxial test experiment data. HY and DT wrote the manuscript.

## Conflict of Interest

The authors declare that the research was conducted in the absence of any commercial or financial relationships that could be construed as a potential conflict of interest.

## Abbreviations

CMR: cardiac magnetic resonance
LV: left ventricle
PVI: pulmonary valve insertion
PVR: pulmonary valve replacement
RV: right ventricle
TOF: tetralogy of fallot
YM: young’s modulus
